# Aqua{2-(morpholin-4-yl)-*N*-[1-(2-pyri­dyl)ethyl­idene]ethanamine-κ^3^ *N*,*N*′,*N*′′}bis­(thio­cyanato-κ*N*)manganese(II)

**DOI:** 10.1107/S1600536811022124

**Published:** 2011-06-18

**Authors:** Nura Suleiman Gwaram, Hamid Khaledi, Hapipah Mohd Ali

**Affiliations:** a Department of Chemistry, University of Malaya, 50603 Kuala Lumpur, Malaysia

## Abstract

In the title compound, [Mn(NCS)_2_(C_13_H_19_N_3_O)(H_2_O)], the Schiff base acts as an *N,N′,N"*-tridentate ligand, forming two five-membered chelating rings with the Mn^II^ atom. The distorted octa­hedral geometry around the metal atom is completed by two *cis*-positioned *N*-bound thio­cyanate ligands and one water mol­ecule. In the crystal, adjacent mol­ecules are linked through O—H⋯O, O—H⋯S and C—H⋯S hydrogen bonds into a three-dimensional supra-mol­ecular structure. An intra­molecular C—H⋯O hydrogen bond also occurs.

## Related literature

For the isostructural Co(II) complex, see: Suleiman Gwaram *et al.* (2011 ▶).
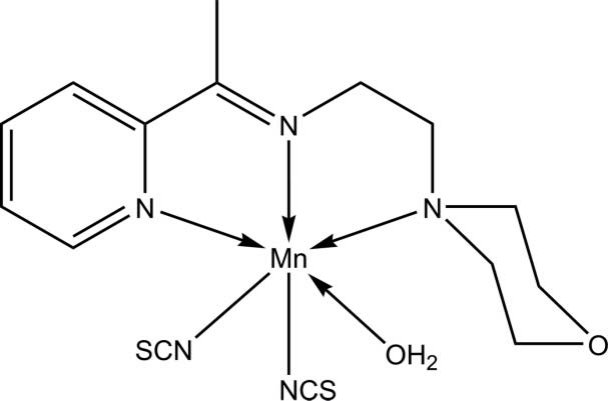


## Experimental

### 

#### Crystal data

[Mn(NCS)_2_(C_13_H_19_N_3_O)(H_2_O)]
*M* *_r_* = 422.43
Monoclinic, 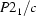

*a* = 7.1837 (13) Å
*b* = 22.408 (4) Å
*c* = 12.112 (2) Å
β = 91.149 (3)°
*V* = 1949.3 (6) Å^3^
*Z* = 4
Mo *K*α radiation
μ = 0.91 mm^−1^
*T* = 100 K
0.19 × 0.16 × 0.08 mm

#### Data collection

Bruker APEXII CCD diffractometer
Absorption correction: multi-scan (*SADABS*; Sheldrick, 1996 ▶) *T*_min_ = 0.846, *T*_max_ = 0.931
11816 measured reflections
4235 independent reflections
3197 reflections with *I* > 2σ(*I*)
*R*_int_ = 0.040

#### Refinement

*R*[*F* ^2^ > 2σ(*F* ^2^)] = 0.037
*wR*(*F* ^2^) = 0.082
*S* = 1.01
4235 reflections
233 parameters
3 restraints
H atoms treated by a mixture of independent and constrained refinement
Δρ_max_ = 0.33 e Å^−3^
Δρ_min_ = −0.46 e Å^−3^

### 

Data collection: *APEX2* (Bruker, 2007 ▶); cell refinement: *SAINT* (Bruker, 2007 ▶); data reduction: *SAINT*; program(s) used to solve structure: *SHELXS97* (Sheldrick, 2008 ▶); program(s) used to refine structure: *SHELXL97* (Sheldrick, 2008 ▶); molecular graphics: *X-SEED* (Barbour, 2001 ▶); software used to prepare material for publication: *SHELXL97* and *publCIF* (Westrip, 2010 ▶).

## Supplementary Material

Crystal structure: contains datablock(s) I, global. DOI: 10.1107/S1600536811022124/xu5237sup1.cif

Structure factors: contains datablock(s) I. DOI: 10.1107/S1600536811022124/xu5237Isup2.hkl

Additional supplementary materials:  crystallographic information; 3D view; checkCIF report

## Figures and Tables

**Table 1:** Selected bond lengths (Å)

Mn1—N1	2.2835 (19)
Mn1—N2	2.2115 (18)
Mn1—N3	2.3891 (19)
Mn1—N4	2.137 (2)
Mn1—N5	2.145 (2)
Mn1—O2	2.2117 (17)

**Table 2:** Hydrogen-bond geometry (Å, °)

*D*—H⋯*A*	*D*—H	H⋯*A*	*D*⋯*A*	*D*—H⋯*A*
O2—H2*A*⋯S1^i^	0.82 (2)	2.38 (2)	3.1910 (18)	169 (2)
O2—H2*B*⋯O1^ii^	0.82 (2)	1.89 (2)	2.693 (2)	164 (3)
C11—H11*A*⋯O2	0.99	2.41	3.179 (3)	134
C12—H12*A*⋯S2^iii^	0.99	2.82	3.674 (2)	145

Symmetry codes: (i) 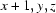; (ii) 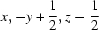; (iii) 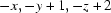.

**Fig. 1.**
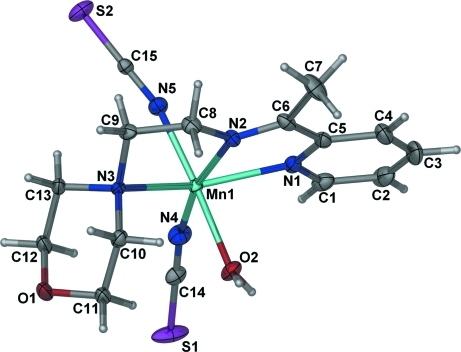
Molecular structure of the title compound with thermal ellipsoids at the 50% probability level. Hydrogen atoms are drawn as spheres of arbitrary radius.

## supplementary crystallographic information

### Comment

The crystal of the title compound is isostructural with the previously reported Co^II^ complex (Suleiman Gwaram *et al.*, 2011). The metal center is coordinated by the *N,N',N"*-tridentate Schiff base, two *N*-donor thiocyanates and one water O atom in a distorted octahedral geometry. In the crystal, the molecules are linked through O—H···O and O—H···S hydrogen bonds into layers parallel to the *ac* plane and theses are connected into a three-dimensional network *via* C—H···S interactions. Moreover, intramolecular C—H···O hydrogen bonding is observed.

### Experimental

A mixture of 2-acetylpyridine (0.20 g, 1.65 mmol) and 4-(2-aminoethyl)morpholine (0.21 g, 1.65 mmol) in ethanol (20 ml) was refluxed for 2 hr followed by addition of a solution of manganese(II) acetate tetrahydrate (0.41 g, 1.65 mmol) and ammonium thiocyanate (0.13 g, 1.65 mmol) in a minimum amount of ethanol. The resulting solution was refluxed for 30 min, and then left at room temperature. The crystals of the title complex were obtained in a week.

### Refinement

The C-bound H atoms were placed at calculated positions and were treated as riding on their parent C atoms with C—H = 0.95 (aryl), 0.98 (methyl) and 0.99 (methylene) Å. The O-bound H atoms were located in a difference Fourier map, and refined with a distance restraint of O–H 0.84±0.02. For all H atoms, *U*_iso_(H) was set to 1.2(1.5 for methyl)*U*_eq_(carrier atom). An additional rigid-bond type restraint (DELU in *SHELXL97*) was placed on the displacement parameters of S2 and C15.

### Crystal data

**Table:**

[Mn(NCS)_2_(C_13_H_19_N_3_O)(H_2_O)]	*F*(000) = 876
*M**_r_* = 422.43	*D*_x_ = 1.439 Mg m^−^^3^
Monoclinic, *P*2_1_/*c*	Mo *K*α radiation, λ = 0.71073 Å
Hall symbol: -P 2ybc	Cell parameters from 2405 reflections
*a* = 7.1837 (13) Å	θ = 2.5–27.0°
*b* = 22.408 (4) Å	µ = 0.91 mm^−^^1^
*c* = 12.112 (2) Å	*T* = 100 K
β = 91.149 (3)°	Prismatic, brown
*V* = 1949.3 (6) Å^3^	0.19 × 0.16 × 0.08 mm
*Z* = 4	

### Data collection

**Table:**

Bruker APEXII CCD diffractometer	4235 independent reflections
Radiation source: fine-focus sealed tube	3197 reflections with *I* > 2σ(*I*)
graphite	*R*_int_ = 0.040
φ and ω scans	θ_max_ = 27.0°, θ_min_ = 1.8°
Absorption correction: multi-scan (*SADABS*; Sheldrick, 1996)	*h* = −9→9
*T*_min_ = 0.846, *T*_max_ = 0.931	*k* = −28→15
11816 measured reflections	*l* = −13→15

### Refinement

**Table:**

Refinement on *F*^2^	Primary atom site location: structure-invariant direct methods
Least-squares matrix: full	Secondary atom site location: difference Fourier map
*R*[*F*^2^ > 2σ(*F*^2^)] = 0.037	Hydrogen site location: inferred from neighbouring sites
*wR*(*F*^2^) = 0.082	H atoms treated by a mixture of independent and constrained refinement
*S* = 1.01	*w* = 1/[σ^2^(*F*_o_^2^) + (0.0302*P*)^2^ + 0.8463*P*] where *P* = (*F*_o_^2^ + 2*F*_c_^2^)/3
4235 reflections	(Δ/σ)_max_ = 0.010
233 parameters	Δρ_max_ = 0.33 e Å^−^^3^
3 restraints	Δρ_min_ = −0.46 e Å^−^^3^

### Special details

**Table:**

Geometry. All e.s.d.'s (except the e.s.d. in the dihedral angle between two l.s. planes) are estimated using the full covariance matrix. The cell e.s.d.'s are taken into account individually in the estimation of e.s.d.'s in distances, angles and torsion angles; correlations between e.s.d.'s in cell parameters are only used when they are defined by crystal symmetry. An approximate (isotropic) treatment of cell e.s.d.'s is used for estimating e.s.d.'s involving l.s. planes.
Refinement. Refinement of *F*^2^ against ALL reflections. The weighted *R*-factor *wR* and goodness of fit *S* are based on *F*^2^, conventional *R*-factors *R* are based on *F*, with *F* set to zero for negative *F*^2^. The threshold expression of *F*^2^ > σ(*F*^2^) is used only for calculating *R*-factors(gt) *etc*. and is not relevant to the choice of reflections for refinement. *R*-factors based on *F*^2^ are statistically about twice as large as those based on *F*, and *R*- factors based on ALL data will be even larger.

### Fractional atomic coordinates and isotropic or equivalent isotropic displacement parameters (Å^2^)

**Table:**

	*x*	*y*	*z*	*U*_iso_*/*U*_eq_	
Mn1	0.32353 (4)	0.376455 (15)	0.71236 (3)	0.01469 (10)	
S1	−0.15046 (9)	0.22409 (3)	0.71859 (8)	0.0455 (2)	
S2	0.08739 (9)	0.56599 (3)	0.84034 (6)	0.03342 (18)	
O1	0.3684 (2)	0.29611 (7)	1.04053 (13)	0.0274 (4)	
O2	0.4538 (2)	0.28777 (8)	0.69253 (14)	0.0240 (4)	
H2A	0.562 (3)	0.2763 (11)	0.701 (2)	0.029*	
H2B	0.406 (3)	0.2628 (10)	0.6508 (19)	0.029*	
N1	0.3511 (2)	0.38231 (8)	0.52518 (15)	0.0193 (4)	
N2	0.5890 (2)	0.42204 (8)	0.67754 (15)	0.0167 (4)	
N3	0.4725 (2)	0.38883 (8)	0.88902 (15)	0.0159 (4)	
N4	0.0713 (3)	0.32585 (10)	0.71154 (18)	0.0291 (5)	
N5	0.1827 (3)	0.45968 (9)	0.73672 (16)	0.0216 (4)	
C1	0.2350 (3)	0.35789 (11)	0.4504 (2)	0.0263 (6)	
H1	0.1197	0.3424	0.4745	0.032*	
C2	0.2752 (4)	0.35409 (12)	0.3394 (2)	0.0341 (6)	
H2	0.1883	0.3371	0.2883	0.041*	
C3	0.4436 (4)	0.37543 (13)	0.3045 (2)	0.0375 (7)	
H3	0.4762	0.3724	0.2291	0.045*	
C4	0.5648 (4)	0.40130 (12)	0.3805 (2)	0.0305 (6)	
H4	0.6811	0.4167	0.3578	0.037*	
C5	0.5147 (3)	0.40447 (10)	0.49019 (19)	0.0199 (5)	
C6	0.6389 (3)	0.43017 (11)	0.57811 (19)	0.0206 (5)	
C7	0.8112 (3)	0.46256 (13)	0.5449 (2)	0.0345 (7)	
H7A	0.8537	0.4885	0.6054	0.052*	
H7B	0.7838	0.4868	0.4791	0.052*	
H7C	0.9089	0.4336	0.5283	0.052*	
C8	0.6995 (3)	0.44329 (11)	0.77211 (19)	0.0217 (5)	
H8A	0.7483	0.4837	0.7568	0.026*	
H8B	0.8065	0.4163	0.7858	0.026*	
C9	0.5774 (3)	0.44504 (10)	0.87324 (19)	0.0197 (5)	
H9A	0.6566	0.4526	0.9396	0.024*	
H9B	0.4882	0.4785	0.8656	0.024*	
C10	0.6016 (3)	0.34062 (10)	0.92493 (19)	0.0209 (5)	
H10A	0.6755	0.3543	0.9900	0.025*	
H10B	0.6889	0.3319	0.8649	0.025*	
C11	0.4980 (3)	0.28452 (11)	0.95414 (19)	0.0250 (6)	
H11A	0.4299	0.2694	0.8880	0.030*	
H11B	0.5877	0.2535	0.9788	0.030*	
C12	0.2375 (3)	0.34069 (11)	1.0051 (2)	0.0245 (5)	
H12A	0.1470	0.3480	1.0642	0.029*	
H12B	0.1681	0.3265	0.9388	0.029*	
C13	0.3372 (3)	0.39803 (10)	0.97860 (19)	0.0209 (5)	
H13A	0.2450	0.4286	0.9555	0.025*	
H13B	0.4037	0.4128	1.0456	0.025*	
C14	−0.0226 (3)	0.28415 (11)	0.7138 (2)	0.0227 (5)	
C15	0.1420 (3)	0.50395 (10)	0.78071 (18)	0.0158 (4)	

### Atomic displacement parameters (Å^2^)

**Table:**

	*U*^11^	*U*^22^	*U*^33^	*U*^12^	*U*^13^	*U*^23^
Mn1	0.01428 (16)	0.01413 (18)	0.01570 (18)	−0.00171 (13)	0.00136 (12)	−0.00224 (14)
S1	0.0221 (3)	0.0171 (3)	0.0974 (7)	−0.0034 (3)	0.0035 (4)	−0.0039 (4)
S2	0.0328 (3)	0.0233 (4)	0.0442 (4)	0.0049 (3)	0.0016 (3)	−0.0134 (3)
O1	0.0381 (10)	0.0239 (10)	0.0206 (9)	0.0099 (8)	0.0089 (7)	0.0075 (7)
O2	0.0243 (9)	0.0218 (10)	0.0255 (10)	0.0047 (7)	−0.0053 (7)	−0.0081 (7)
N1	0.0207 (9)	0.0191 (11)	0.0179 (10)	0.0029 (8)	−0.0024 (8)	−0.0006 (8)
N2	0.0151 (8)	0.0190 (11)	0.0159 (10)	−0.0024 (7)	−0.0013 (7)	0.0015 (8)
N3	0.0214 (9)	0.0126 (10)	0.0137 (10)	0.0024 (7)	0.0012 (7)	−0.0006 (8)
N4	0.0211 (10)	0.0268 (13)	0.0394 (13)	−0.0052 (9)	0.0034 (9)	−0.0048 (10)
N5	0.0197 (9)	0.0214 (11)	0.0238 (11)	0.0021 (8)	−0.0002 (8)	−0.0008 (9)
C1	0.0291 (13)	0.0203 (14)	0.0291 (15)	0.0032 (10)	−0.0088 (11)	−0.0027 (11)
C2	0.0542 (17)	0.0258 (15)	0.0216 (15)	0.0046 (13)	−0.0162 (13)	−0.0041 (11)
C3	0.0604 (19)	0.0378 (17)	0.0142 (13)	0.0062 (14)	0.0003 (12)	0.0011 (12)
C4	0.0399 (15)	0.0308 (15)	0.0211 (14)	0.0036 (12)	0.0053 (11)	0.0064 (11)
C5	0.0235 (11)	0.0179 (13)	0.0182 (12)	0.0043 (9)	0.0006 (9)	0.0035 (10)
C6	0.0180 (10)	0.0217 (13)	0.0224 (13)	0.0016 (9)	0.0011 (9)	0.0076 (10)
C7	0.0211 (12)	0.0511 (19)	0.0314 (16)	−0.0071 (12)	0.0020 (11)	0.0174 (13)
C8	0.0202 (11)	0.0221 (13)	0.0225 (13)	−0.0067 (9)	−0.0040 (9)	0.0006 (10)
C9	0.0249 (11)	0.0155 (12)	0.0185 (13)	−0.0034 (9)	−0.0067 (9)	−0.0020 (9)
C10	0.0238 (11)	0.0203 (13)	0.0187 (13)	0.0043 (10)	−0.0001 (9)	−0.0017 (10)
C11	0.0370 (13)	0.0202 (14)	0.0181 (13)	0.0080 (11)	0.0065 (11)	0.0040 (10)
C12	0.0318 (13)	0.0247 (14)	0.0175 (13)	0.0078 (10)	0.0075 (10)	0.0062 (10)
C13	0.0302 (12)	0.0186 (13)	0.0140 (12)	0.0069 (10)	0.0038 (10)	0.0008 (9)
C14	0.0154 (10)	0.0222 (14)	0.0306 (14)	0.0023 (10)	0.0007 (10)	−0.0049 (11)
C15	0.0137 (10)	0.0179 (10)	0.0158 (12)	0.0012 (8)	−0.0006 (8)	0.0028 (8)

### Geometric parameters (Å, °)

**Table:**

Mn1—N1	2.2835 (19)	C3—C4	1.382 (4)
Mn1—N2	2.2115 (18)	C3—H3	0.9500
Mn1—N3	2.3891 (19)	C4—C5	1.386 (3)
Mn1—N4	2.137 (2)	C4—H4	0.9500
Mn1—N5	2.145 (2)	C5—C6	1.491 (3)
Mn1—O2	2.2117 (17)	C6—C7	1.497 (3)
S1—C14	1.631 (2)	C7—H7A	0.9800
S2—C15	1.618 (2)	C7—H7B	0.9800
O1—C12	1.432 (3)	C7—H7C	0.9800
O1—C11	1.438 (3)	C8—C9	1.521 (3)
O2—H2A	0.820 (16)	C8—H8A	0.9900
O2—H2B	0.824 (16)	C8—H8B	0.9900
N1—C1	1.336 (3)	C9—H9A	0.9900
N1—C5	1.352 (3)	C9—H9B	0.9900
N2—C6	1.276 (3)	C10—C11	1.507 (3)
N2—C8	1.460 (3)	C10—H10A	0.9900
N3—C9	1.482 (3)	C10—H10B	0.9900
N3—C10	1.483 (3)	C11—H11A	0.9900
N3—C13	1.486 (3)	C11—H11B	0.9900
N4—C14	1.153 (3)	C12—C13	1.509 (3)
N5—C15	1.166 (3)	C12—H12A	0.9900
C1—C2	1.384 (4)	C12—H12B	0.9900
C1—H1	0.9500	C13—H13A	0.9900
C2—C3	1.375 (4)	C13—H13B	0.9900
C2—H2	0.9500		
			
N4—Mn1—N5	93.43 (8)	N2—C6—C5	116.3 (2)
N4—Mn1—N2	168.07 (8)	N2—C6—C7	124.9 (2)
N5—Mn1—N2	92.03 (7)	C5—C6—C7	118.8 (2)
N4—Mn1—O2	83.30 (7)	C6—C7—H7A	109.5
N5—Mn1—O2	176.33 (7)	C6—C7—H7B	109.5
N2—Mn1—O2	91.49 (7)	H7A—C7—H7B	109.5
N4—Mn1—N1	96.67 (8)	C6—C7—H7C	109.5
N5—Mn1—N1	97.88 (7)	H7A—C7—H7C	109.5
N2—Mn1—N1	72.05 (7)	H7B—C7—H7C	109.5
O2—Mn1—N1	84.17 (7)	N2—C8—C9	109.09 (17)
N4—Mn1—N3	115.48 (7)	N2—C8—H8A	109.9
N5—Mn1—N3	88.85 (7)	C9—C8—H8A	109.9
N2—Mn1—N3	75.21 (6)	N2—C8—H8B	109.9
O2—Mn1—N3	91.07 (6)	C9—C8—H8B	109.9
N1—Mn1—N3	146.75 (7)	H8A—C8—H8B	108.3
C12—O1—C11	109.80 (17)	N3—C9—C8	112.62 (18)
Mn1—O2—H2A	132.0 (19)	N3—C9—H9A	109.1
Mn1—O2—H2B	120.5 (19)	C8—C9—H9A	109.1
H2A—O2—H2B	104 (3)	N3—C9—H9B	109.1
C1—N1—C5	118.2 (2)	C8—C9—H9B	109.1
C1—N1—Mn1	125.76 (16)	H9A—C9—H9B	107.8
C5—N1—Mn1	115.18 (14)	N3—C10—C11	111.58 (19)
C6—N2—C8	122.36 (19)	N3—C10—H10A	109.3
C6—N2—Mn1	120.36 (15)	C11—C10—H10A	109.3
C8—N2—Mn1	117.27 (13)	N3—C10—H10B	109.3
C9—N3—C10	109.89 (17)	C11—C10—H10B	109.3
C9—N3—C13	108.54 (17)	H10A—C10—H10B	108.0
C10—N3—C13	107.53 (17)	O1—C11—C10	110.50 (19)
C9—N3—Mn1	101.71 (12)	O1—C11—H11A	109.6
C10—N3—Mn1	116.38 (13)	C10—C11—H11A	109.6
C13—N3—Mn1	112.49 (14)	O1—C11—H11B	109.6
C14—N4—Mn1	157.83 (19)	C10—C11—H11B	109.6
C15—N5—Mn1	157.80 (18)	H11A—C11—H11B	108.1
N1—C1—C2	123.0 (2)	O1—C12—C13	110.3 (2)
N1—C1—H1	118.5	O1—C12—H12A	109.6
C2—C1—H1	118.5	C13—C12—H12A	109.6
C3—C2—C1	118.6 (2)	O1—C12—H12B	109.6
C3—C2—H2	120.7	C13—C12—H12B	109.6
C1—C2—H2	120.7	H12A—C12—H12B	108.1
C2—C3—C4	119.2 (2)	N3—C13—C12	110.95 (18)
C2—C3—H3	120.4	N3—C13—H13A	109.4
C4—C3—H3	120.4	C12—C13—H13A	109.4
C3—C4—C5	119.2 (2)	N3—C13—H13B	109.4
C3—C4—H4	120.4	C12—C13—H13B	109.4
C5—C4—H4	120.4	H13A—C13—H13B	108.0
N1—C5—C4	121.8 (2)	N4—C14—S1	178.4 (2)
N1—C5—C6	115.45 (19)	N5—C15—S2	179.1 (2)
C4—C5—C6	122.8 (2)		

### Hydrogen-bond geometry (Å, °)

**Table:**

*D*—H···*A*	*D*—H	H···*A*	*D*···*A*	*D*—H···*A*
O2—H2A···S1^i^	0.82 (2)	2.38 (2)	3.1910 (18)	169 (2)
O2—H2B···O1^ii^	0.82 (2)	1.89 (2)	2.693 (2)	164 (3)
C11—H11A···O2	0.99	2.41	3.179 (3)	134
C12—H12A···S2^iii^	0.99	2.82	3.674 (2)	145

Symmetry codes: (i) *x*+1, *y*, *z*; (ii) *x*, −*y*+1/2, *z*−1/2; (iii) −*x*, −*y*+1, −*z*+2.

## References

[bb1] Barbour, L. J. (2001). *J. Supramol. Chem.* **1**, 189–191.

[bb2] Bruker (2007). *APEX2* and *SAINT* Bruker AXS Inc., Madison, Wisconsin, USA.

[bb3] Sheldrick, G. M. (1996). *SADABS* University of Göttingen, Germany.

[bb4] Sheldrick, G. M. (2008). *Acta Cryst.* A**64**, 112–122. 10.1107/S0108767307043930 18156677

[bb5] Suleiman Gwaram, N., Ikmal Hisham, N. A., Khaledi, H. & Mohd Ali, H. (2011). *Acta Cryst.* E**67**, m108. 10.1107/S1600536810052578 PMC3050266 21522521

[bb6] Westrip, S. P. (2010). *J. Appl. Cryst.* **43**, 920–925.

